# Management of Multiple Myeloma in the Middle East: Unmet Needs, Challenges and Perspective

**DOI:** 10.1007/s44228-022-00017-3

**Published:** 2022-08-30

**Authors:** Ahmad Ibrahim, Nabil Chamseddine, Jean El-cheikh, Colette Hanna, Walid Moukadem, Fady Nasr, Ahmad Younis, Ali Bazarbachi

**Affiliations:** 1 Department of Hematology-Oncology, Makassed University Hospital, Beirut, Lebanon; 2 School of Medicine, National Lebanese University, Hadath, Lebanon; 3 Department of Hematology-Oncology, Middle East Institute of Health, Beirut, Lebanon; 4 Department of Hematology-Oncology, Saint George Hospital University Medical Center, Beirut, Lebanon; 5 Department of Internal Medicine, American University of Beirut Medical Center, Beirut, Lebanon; 6 Department of Hematology-Oncology, Clemenceau Medical Center, Beirut, Lebanon; 7 Department of Hematology-Oncology, Hôtel-Dieu de France Medical Center, Beirut, Lebanon; 8 Department of Hematology-Oncology, Mount Lebanon Hospital, Hazmieh, Lebanon; 9 Department of Hematology-Oncology, Haykel Hospital, Tripoli, Lebanon; 10 Department of Hematology-Oncology, Military Hospital, Beirut, Lebanon

**Keywords:** Multiple myeloma, Disease management, Very elderly, Recurrence, Minimal residual disease, Middle East

## Abstract

Multiple myeloma (MM) is a prevalent hematological malignancy. Resource-constrained settings such as the Middle East are particularly burdened by the increasing trends in MM morbidity and mortality in addition to challenges in the management of MM. It thus becomes necessary to identify and address debatable areas of current practice and gaps in the management of MM in the Middle East. With a special focus on the Lebanese situation, the first-line treatment of the very elderly (> 80 years old) is discussed, in addition to the impact of relapse type (biochemical or clinical relapse) on maintenance therapy, the choice of first relapse therapy in relation to maintenance therapy, and the role of MRD in the MM treatment landscape. The need for realistic management guidelines accounting for local resources and expertise, in addition to the reflection of drug accessibility and cost on clinical practice are recognized.

## Introduction

Multiple myeloma (MM) is one of the most prevalent hematological malignancies, with 176,404 new cases and 117,077 deaths reported globally in 2020 [1]. Resource-constrained settings, such as the Middle East, are particularly beset not only with the globally increasing trend in both MM incidence and mortality, but also with the high socioeconomic burden of the disease [2]. The UAE and Qatar were actually one of the countries with the largest growth in MM cases and deaths over the past three decades across the world [3]. MM treatment targets the control of existing malignancy, the management of disease complications, and the prevention of progression. Despite the advancement in diagnosis and therapies, the treatment of MM, particularly relapsed cases, remains beset with challenges. Evidence currently argues in favor of treatment personalization in MM [4]. While novel drugs and other therapeutic interventions improve the prognosis of MM, treatment accessibility remains limited across the Middle East [5], including Lebanon [6]. In addition to the restrictions of therapy availability, and despite recent national guidelines established by the Lebanese Society of Hematology and Blood Transfusion which were adopted by the Lebanese Ministry of Health, there is a lack of comprehensive data to inform the decision-making process of local patients. For instance, Lebanon faces the lack of epidemiological, etiological, and clinical studies in the context of MM. While scant, published data reveal the increasing trend in MM incidence [7], the poor survival of elderly patients, and the need for less toxic alternative treatment options [8, 9]. With this perspective, areas of current practice that are the focus of considerable debate and constitute a challenge/gap in the management of MM in the Middle East are explored, with a special focus on the Lebanese situation.

## Frontline Therapy: Focus On The Treatment Of The Very Elderly (> 80 Years)

Current real-world evidence shows that triplet therapy followed by autologous hematopoietic stem cell transplantation, consolidation and maintenance for fit responder patients remains the backbone of MM treatment in Lebanon, while novel regimens are being explored whenever possible [9]. Fit patients might even receive quadruplet regimens, while adjusted intensity regimens are optimal for frail elderly patients [10]. The inevitability of aging and its associated comorbidities and biological changes complicate the treatment of very elderly MM patients. These patient populations continue to exhibit poor outcomes and are systematically undertreated, even in developed countries such as the United States [11], in addition to being frequently excluded from clinical trials. With the continuously shifting paradigm of MM management, educational gaps emerge and targeted initiatives are needed to guide oncologists in the personalization of treatment and the translation of evidence-based recommendations into real-world practice [12]. Notwithstanding advanced age, MM patients should be treated, and their management individualized, in order to fulfil goals of care. Regardless, it is evident that frailty carries significant implications for the survival of MM patients, and can be determined in the very elderly using a frailty score [13]. Indeed, the International Myeloma Working Group (IMWG) has developed a frailty score which is calculated to categorize older patients with MM as fit, intermediate fit, or frail. The frailty status is associated with drug discontinuation, increased rates of Grade 3–4 nonhematologic toxicities in addition to worse survival. Based on this score, patients aged over 80 years old are frail [11]. Geriatric assessments should be integrated into routine clinical practice not only for the timely diagnosis of MM and the exclusion of differential diagnoses in the very elderly, but also to allow for more optimal treatment selection and modification of both dosing and regimens to better balance effectiveness and tolerability [10]**.** Dose reduction of novel drugs and schedule adjustment can be beneficial with similar outcomes in unfit patients. Furthermore, in the modern era, several studies have demonstrated the toxicity of long-term use of steroids in non-transplant eligible patients and emphasized the value of decreasing the dose and/or reducing the duration of steroid administration with lower toxicity and mortality [14–16]**.** With the risks of melphalan-based regimens in the very elderly, novel drugs should be considered, if available, either as part of treatment or maintenance therapy. This does not preclude the consideration of older drug regimens, which are also effective as maintenance for elderly MM patients [17]. Front-line treatment with novel drugs are used until disease progression in patients who are not transplant eligible, and maintenance therapy is not considered [18]. However, as with younger patients, maintenance therapy should be continuous in the very elderly. That being said, the treatment of geriatric patient populations experiencing disease relapse remains fraught with challenges and warrants further investigation.

## When To Initiate Relapse Therapy: Biochemical Versus Symptomatic Relapse

The 2021 European Hematology Association (EHA)-European Society for Medical Oncology (ESMO) and the IMWG guidelines offered detailed recommendations for the management of relapsed MM patients [19, 20]. In general, reducing the burden of active symptoms, prolonging progression-free survival and overall survival as well as achieving disease response (e.g. complete remission, minimal residual disease (MRD) negativity) are important goals when treating relapsed MM. Patients should be treated in order to achieve these goals and prevent any additional morbidity while also preserving quality of life. These guidelines mainly focus on prior therapy, its duration and efficacy as well as cytogenetics. The management of relapsing patients should be individualized considering the heterogeneity of MM relapse types and symptoms. However, risk-adapted strategies remain limited to the presence of a translocation t(11;14) in case of availability of venetoclax, as no other factor was recommended for routine risk stratification [19]. It is also important to consider the duration of first remission and whether the disease progressed or relapsed during treatment. Other patient-related and treatment/disease-related factors to consider include patient age, performance status, bone marrow reserve, renal function, pre-existing toxicity (e.g. peripheral neuropathy, thromboembolic events), quality of life, and cost.

However, even with the availability of treatment recommendations, the question remains: which drugs are available and when should relapse therapy be initiated? Drug availability is an important factor and treatment guidelines such as the 2021 IMWG recommendations are progressively making provisions for drug access limitations in low- and middle-income countries [20]. Regarding therapy initiation, it might be important to distinguish between a symptomatic relapse (characterized by the reoccurrence of clinical symptoms, namely calcium elevation, renal dysfunction, anemia, and bone disease (CRAB) criteria) and a biochemical relapse (defined as a 25% increase in the paraprotein from the lowest response value without CRAB symptoms) [21]. MM relapse management largely depends on clinical observation and follow-up, the frequency of which should be determined based on the kinetics of M-protein increase. It is clear that a symptomatic relapse generally indicates the need to initiate treatment. While variability in relapse symptoms can be observed, it is accepted that a symptomatic relapse should be treated if any of the criteria proposed by the IMWG are met [22]. The 2015 IMWG consensus on the second-line treatment of transplant-ineligible patients also indicated the need for treatment initiation upon the detection of a significant and quick paraprotein increase (defined as doubled monoclonal protein within 2 months, with an increase in the absolute levels of monoclonal protein of > 1 g/dL in serum or of > 500 mg per 24 h in urine, confirmed by two consecutive measurements), regardless of the presence of clinical symptoms of disease activity [21]. A more dismal prognosis is evidenced in patients with clinical progression and those who receive treatment at this stage as opposed to those receiving early treatment at signs of biochemical progression [23, 24]. Pre-emptive treatment initiation in patients with biochemical relapse before the onset of end-organ damage could thus be beneficial [25]. However, this approach remains debatable, and clinicians continue to face the “not too early, not too late” dilemma. To that end, it is important to identify local indicators or risk factors (e.g. aggressive clinical disease at diagnosis, short treatment-free interval with a suboptimal response to the previous treatment line, unfavorable cytogenetics: t(4;14) or deletion del(17p)), which might justify treatment initiation in asymptomatic patients exhibiting clear signs of a biochemical relapse [26]. While optimizing treatment timing carries prognostic implications, it is also important to distinguish oligoclonal reconstitution (which results in measurable M-protein particularly after autologous stem cell transplantation (ASCT) [27] from significant biochemical relapse in order to avoid overtreatment.

## Impact of Maintenance After First-Line Therapy on Choice of First-Relapse Therapy

Younger, fit patients who are eligible for transplant should be considered for ASCT, seeing as it remains one of the most effective and widely available therapies for MM. Tandem ASCT as consolidation can also be considered only for high-risk cytogenetic patients [28]. Maintenance therapy should be provided post-ASCT. Continuous frontline therapy translates into delaying initial disease progression and overall survival, in addition to clinical benefit after the first relapse (prolonged progression-free survival until the second relapse) [22]. Treatment should be continued until maximum response is achieved, after which maintenance should be considered until progression or a lack of tolerability. Achieving MRD negativity is an important treatment goal, but the risk of iatrogenic side effects (e.g. cytopenia, rashes, and peripheral neuropathy) should be carefully considered. Nevertheless, real-world patterns of MM treatment and diagnosis are often quite different from published evidence-based recommendations, even in developed countries [23]. This disparity is particularly striking in low-resource settings [24]. The prohibitive cost of MM treatment, which often involves two or more agents, and the limitations to drug access, emphasize the need to determine both the setting in which each drug regimen provides maximum effectiveness, and the minimal duration of therapy needed to achieve satisfactory durable clinical response [25]. Moreover, access to cytogenetic services (i.e. fluorescence in situ hybridization and next-generation sequencing), remains limited across countries in the Middle East [33–35]. While it is undeniable that novel drugs significantly improve MM outcomes, physicians in resource-constrained countries, such as Lebanon, continue to struggle to provide even older drugs (e.g. bortezomib and lenalidomide) for their patients. Clinicians should thus be mindful of the depth of response to treatment, and understand its implications as well as those of maintenance therapy with respect to patient outcomes. When selecting treatment for relapsed/refractory MM, it is important to remember that the duration of initial response defines disease biology. Clinicians should also consider disease risk, performance status, age, comorbidities, sensitivity to prior treatment as well as prior and residual toxicities when selecting relapse therapy and optimal dosage [13]. Risk assessment at each relapse is preferable and can be done through biopsies in the absence of more sophisticated methods. As attempted in Saudi Arabia [26] and Asia [27], physicians should adapt national treatment guidelines, while being mindful of locally available drugs and diagnostic capacities in order to continuously provide the most efficacious and cost-effective treatment for patients afflicted with this near incurable malignancy.

## Achieving a Deep Durable Response: MRD Assessment in Myeloma

MRD negativity has notable prognostic implications [28], and should be achieved and maintained in MM patients. In addition to being associated with treatment outcomes, MRD assessment can help discriminate chemoresistance and risk of early progression after transplant [29, 30]. The use of MRD has overcome previous limitations to the setting of clinical trials and has found its way into obligatory recommendations for the evaluation of treatment response and follow-up [13]. MRD assessment can be undertaken through several methods, including the standard serum/urine protein electrophoresis and serum free light-chain analysis. However, these tests have limited sensitivity when disease levels are low. Considering the dramatic increase in complete remission rates among MM populations, alternative options are needed to measure MRD in patients who have achieved a complete response to treatment. Bone marrow analysis using flow cytometry and next-generation sequencing are currently the most relevant and reliable methods for MRD assessment. While more advanced, these techniques are costly and are not readily available across the globe, sometimes extending beyond the capacity of even developed countries [23]. Despite promising data on MRD’s prognostic value, clear definitions of time to MRD and duration of MRD need to be established, in addition to the optimal method for MRD assessment. The potential role of MRD-negative status as an outcome measure influencing local treatment decisions should also be explored. More data are needed to evaluate the association between MRD negativity and positron emission tomography-computed tomography (PET-CT), which would be convenient to use for MRD assessment as PET-CT is a powerful prognostic tool for newly diagnosed MM [40], is available in most settings and has dramatically improved the prognosis of MM. Clinical experience shows that the best overall survival seems to be achieved in patients with standard risk fluorescence in situ hybridization and MRD negativity [41], and the most complicated disease course is seen in patients with PET positivity and MRD negativity. Regardless, early achievement of MRD negativity and its sustainability ensure better survival in MM patients. Its integration into local practice could therefore prove cost-effective in the long-term, by improving MM prognosis and, thereby, reducing the number or the intensity of required therapies.

## Conclusions

When treating MM, patient-related and treatment/disease-related risk factors should be carefully considered, and the management of patients should be individualized accordingly. Gaps in oncologist knowledge and education should be addressed to aid in the personalization of treatment and the integration of evidence-based recommendations into real-world practice. Official, clear and updated guidelines are needed to guide the management of MM patients in Lebanon. Treatment initiation in patients with biochemical relapse without end-organ involvement might be beneficial but remains debatable. The combination of novel agents with standard of care is promising for the induction, consolidation, and maintenance of MM patients. Nevertheless, accessibility and cost should be carefully considered, and realistic management guidelines are needed to optimize diagnosis and treatment efficacy according to local resources. Very elderly patients should not be denied treatment, and adjusted intensity regimens can be used in case of frailty. Achieving MRD negativity is an important treatment goal and carries significant prognostic value. The potential role of MRD-negative status as an outcome measure influencing treatment decisions in local populations, and the feasibility and cost-effectiveness of MRD assessment in everyday practice remains to be explored in the Middle East.
